# Pandemics, vulnerability, and prevention: time to fundamentally reassess how we value and communicate risk? CITIES, HEALTH and COVID-19: Initial reflections and future challenges

**DOI:** 10.1080/23748834.2020.1811480

**Published:** 2020-09-22

**Authors:** Daniel Black, Geoff Bates, Andy Gibson, Eli Hatleskog, Eleonora Fichera, Jenny Hatchard, Hasan Md Nazmul, Ges Rosenberg, Charles Larkin, Rachel Brierley, Judi Kidger, Krista Bondy, Matt Hickman, Kathy Pain, Ben Hicks, Gabriel Scally, Arpana Verma, Neil Carhart, Paul Pilkington, Alistair Hunt, Paddy Ireland

**Affiliations:** a University of Bristol, Bristol, UK; b University of Bath, Bath, UK; c UWE Bristol; d Trinity College Dublin, Johns Hopkins University; e University of Reading, Reading, UK; f University of Manchester, Manchester, UK

**Keywords:** Non-communicable disease, cities, inequality, vulnerability, risk, priorities

## Abstract

For over a decade, pandemics have been on the UK National Risk Register as both the likeliest and most severe of threats. Non-infectious ‘lifestyle’ diseases were already crippling our healthcare services and our economy. COVID-19 has exposed two critical vulnerabilities: firstly, the UK’s failure to adequately assess and communicate the severity of non-communicable disease; secondly, the health inequalities across our society, due not least to the poor quality of our urban environments. This suggests a potentially disastrous lack of preventative action and risk management more generally, notably with regards to the existential risks from the climate and ecological crises.

*‘Vulnerability is the birthplace of innovation and creativity’*

Professor Brené Brown,TED Speaker | ‘The Power of Vulnerability’

For over a decade, pandemics have been recognised on the UK National Risk Register as both the likeliest and most severe of threats, just ahead of flooding, severe weather, and terrorism. Yet we are likely to be among the worst hit of OECD nations in terms of both mortality rate as well as economic fallout (OECD 2020). We now know that those with underlying health conditions are more likely to become severely ill from COVID-19. This has thrown a spotlight on two critical vulnerabilities: firstly, the UK’s failure to adequately assess and communicate the severity of risk from non-communicable disease (NCD); secondly, the stark differences in health and socio-economic status across our society, not least in the quality of the places in which we live in and to which have access (Marmot *et al*. 2010). These failures suggest a potentially disastrous lack of risk management more generally, and should raise significant concerns with regards to the longer-term, existential threats from the climate and ecological crises. There are numerous causal factors at play, and action is needed on multiple fronts, but effective risk management, including risk perception and communication, is arguably the most important.

**Figure 1.**
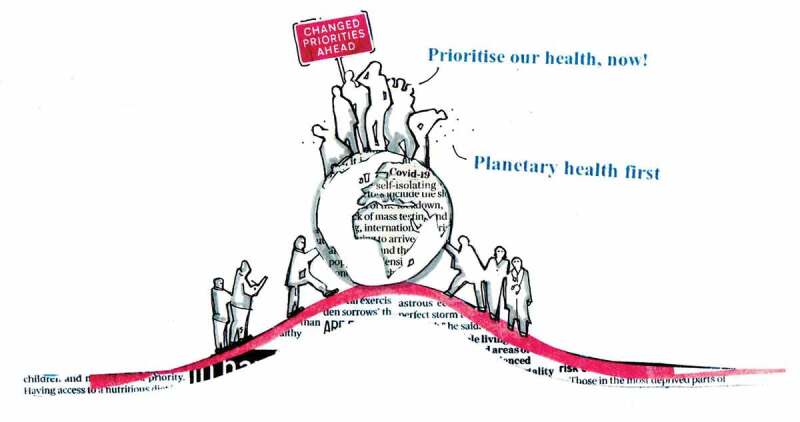
Government intervention as a result of COVID-19 presents a tipping point in terms of our future quality of life.Image credit: Eli Hatleskog

In the UK, NCDs such as heart failure, cancer, obesity, and mental ill-health cause an estimated 89% of deaths, and significant-associated costs from treating morbidity (e.g. obesity alone costs the NHS £5bn per year and an estimated £27bn per year due to its effects on productivity, earnings, and welfare payments; the NHS requires another £5.5bn per year to treating illness from smoking, alcohol, and mental health) (Gov 2017). Climate and ecological impacts, which are linked to NCDs, are likely to dwarf these costs. Medical care is the largest cost facing UK taxpayers, making up almost 10% (c.£200bn) of total annual Government spending; while investment in prevention is marginal (Office for National Statistics 2017). Cardiovascular disease and cancers alone cost the EU economy €200 bn annually in lost productivity from patients and, significantly, their carers. Yet NCDs, it is widely suggested even within Government, are ‘*to a significant extent, preventable, and the costs … largely avoidable*’ (World Health Organization 2014).

The quality of our urban environments plays a significant role in determining our health, both mental and physical. It is no surprise that air quality could well be a factor in COVID-19-related deaths (Wu and Nethery 2020). It’s also important to recognise however that we may have far more data on air pollution than any other risk factor; noise and lack of access to nature in cities also have significant impacts on our physical and mental health (Eaton *et al*. 2020). In dense, internationally connected cities, risk of transfer of infectious disease is exacerbated through rapid population flows, and lack of quality public space.

One of the greatest challenges is the burden of disease experienced by the least well off, where pre-existing health conditions are most prevalent and the burden to society greatest (Marmot *et al*. 2020). Yet the pandemic has taught us that people in low status, low paid jobs are undervalued and yet essential to the efficient functioning of our society. Men living in England’s most deprived areas live on average almost 10 years less than those from the least deprived, and the respective rate of deaths involving COVID-19 are double. The pandemic, or more specifically the subsequent lockdown, dramatically magnified the role of our living environments in determining health; we now appreciate the importance of space at home and the quality of accessible public space, and we acknowledge the compound impact of loneliness and risk of abuse.

Prevention is now enshrined in health policy – in theory, if not in practice – but the NHS is not (and cannot be) responsible for upstream decisions in other sectors, which are causing these downstream vulnerabilities. This weakness is created by political and economic decisions actively prioritising short-term gains and consumption-based growth over long-term health, equality, and sustainability (Figure 1). The role of government in perpetuating this cannot be denied given the types of activities it chooses to subsidise: e.g. global subsidies of fossil fuels, which contribute the most to both air pollution and climate change, are estimated to be 4.7 USD trillion (6.3% of global GDP), and the UK is the lead culprit within the European Union (International Monetary Fund 2019).

While it is undeniable that certain segments of the private sector are putting the brakes on progress in this area, others are in fact highly proactive, particularly those with interests that may be devalued by impending pressures: e.g. water companies facing drought, investment companies facing stranded assets, reinsurance companies underwriting risk, asset managers reliant on long-term rental incomes. It is to these people and institutions that Mark Carney, former Governor of the Bank of England, was speaking when talking of the ‘tragedy of the horizon’: the idea that national governments and policy-makers are proving themselves powerless to share resources nor think long term for the common good (Bank of England 2015).

Cities are the engines of the global economy, contributing 60% of global GDP, as well as 70% of global CO_2_ emissions and most of the world’s resource consumption. Cities are home to an increasing majority of the global population and are on the frontline facing these threats. Many are trying to address them, yet the resources and powers at their disposal in the UK are limited following years of austerity and continuing centralisation of powers in Whitehall (Global Government Forum 2015). Some have land they control, and in theory they can choose to exercise policies robustly in ways that take public and planetary health into account, but development is largely determined by remote, private sector agencies and mechanisms – shareholder returns, global flows of investment and private sector activity linked to national government growth targets – which are not responsible for the public’s health, equality, or environmental sustainability.

The benefits of assessing, communicating, and managing these risks cannot be overstated. Doing so would not only empower us, allow us to be healthy and increase productivity, but also help us become less vulnerable to future shocks. There will be enormous pressure from vested interests to resume business as usual after lockdown, yet history teaches us too that crises can lead to profound and positive change too (Stark 2017, Wright and Nyberg 2017).

This should be non-party political. Until recently in the UK, there was an All-Party Parliamentary Group devoted to ‘Health in all Policies’. There is another looking at the ‘Limits to Growth’, and there has been the recent Inquiry by the Environmental Audit Committee into Planetary Health. Yet little is shifting. At the top of the UK government, our health and the future of our planet appear to be peripheral, disconnected, and compartmentalised: health means healthcare; environment means farming and the countryside; urban development means housing numbers not quality of place. Short-term thinking and partial economic measures dominate. Partnerships between cities and forward-thinking private sector partners are already seeking to leapfrog inactive central governments, but there is only so much they can do.

Where decision-makers need help, and where future research might have impact, is not downstream in the generation of yet more evidence of the problem, but upstream in terms of how we transition: How can we comprehensively value and manage socio-environmental risk? How can we prioritise public and planetary health? How can we support and enable structural and institutional transitions? How can we rebalance public and private sector to a healthier equilibrium? And, perhaps most importantly, how can the public support the prioritisation and enactment of transition?

Senior decision-makers from both public and private sector agree that health is not adequately accounted for in urban planning and development, and they support the use of non-market economic valuation in helping us to get a sense of the scale of these challenges and to prioritise action; multiple actions have been identified, and on multiple levels, but much is happening already and there is considerable will to shift and shift radically from all sections of society (Black *et al*. 2020).

The response to the pandemic offers a glimpse of what is both possible when political and social will are aligned. We have a small window for action, and there is no rational alternative. When vulnerable, as we are now, we are forced to acknowledge what has weakened us, and to fundamentally reassess our priorities. Surely now is the time to properly acknowledge and communicate these risks, and embed the prevention of current and future ill-health into the national psyche?
